# Single‐cell isogrowth profiling: Uniform inhibition uncovers non‐uniform drug responses

**DOI:** 10.1002/ctm2.1005

**Published:** 2022-07-31

**Authors:** Martin Lukačišin, Adriana Espinosa‐Cantú, Tobias Bollenbach

**Affiliations:** ^1^ Rappaport Faculty of Medicine Technion Haifa Israel; ^2^ Technion Integrated Cancer Center Technion Haifa Israel; ^3^ Institute for Biological Physics University of Cologne Cologne Germany; ^4^ Center for Data and Simulation Science University of Cologne Cologne Germany

1

Non‐genetic inter‐cellular heterogeneity, in which cells from a population sharing the same genome exhibit phenotypic variability, is increasingly recognised as a major variable affecting clinical outcomes. Whether in bacterial infections^1^, ^2^ or malignant tumours,^3^ a small subpopulation of persister cells capable of escaping the therapeutic intervention can eventually negate any short‐term successes of the therapy. Characterising the causes and mechanisms of non‐genetic heterogeneity in cell populations, particularly that elicited by clinical interventions, is thus critical for advancing therapies.

**FIGURE 1:**
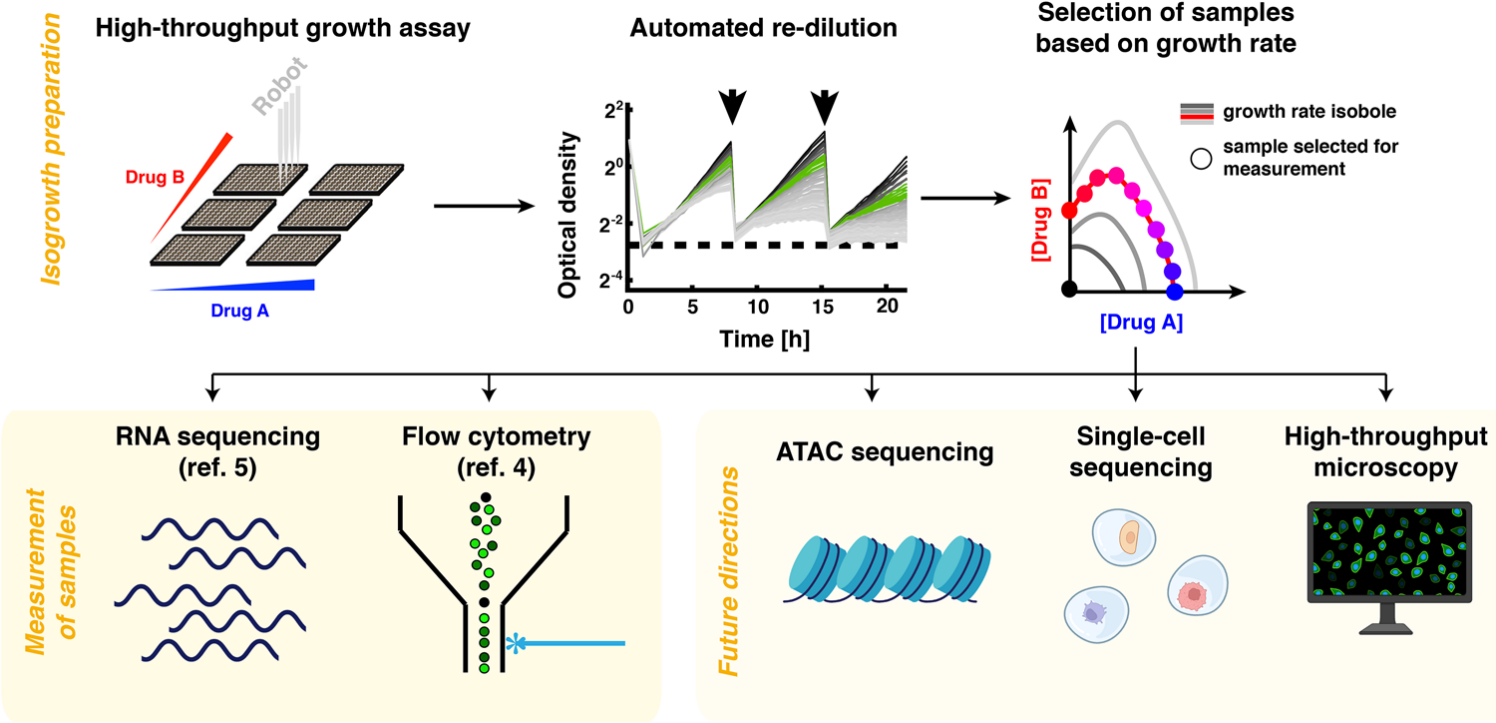
Schematic of the overall design of isogrowth profiling. Samples prepared using isogrowth profiling can be analysed using a variety of methods; examples of possible future directions are shown alongside with approaches that have already been implemented. Adapted from Refs. ^4^ and ^5^ under CC‐BY 4.0 licence; parts of this figure were created with BioRender.com

We recently developed single‐cell isogrowth profiling, an approach to identify and measure non‐genetic heterogeneity induced by drug treatment.^4^ A major source of inaccuracy in the study of responses to chemical interventions is that, in addition to the drug‐specific response, there is a non‐specific effect of stress and slowed growth. In isogrowth profiling, a combination of two drugs is precisely titrated in different ratios to always achieve the same overall growth inhibition in the target population^5^ (Figure 1). In this way, the confounding effect of slower growth is experimentally taken out of the equation: the non‐specific effects of stress and growth inhibition, which are constant across the conditions, can be decoupled from the specific effects of the individual drugs and their combination. Even in research aimed at investigating the effects of a single drug, the addition of another inhibitor for isogrowth profiling plays an indispensable role in modulating growth and detecting quantitative effects of the drug that may manifest only at a certain dose or at a certain growth rate of the target cell. In the single‐cell version of isogrowth profiling, cells cultured using this approach are subsequently characterised using a single‐cell technique, allowing the quantification of heterogeneity. The potential use of a variety of detection methods, bulk or single‐cell, would enable observation of different phenotypes and mechanisms of non‐genetic heterogeneity (Figure 1).

In our pilot study using single‐cell isogrowth profiling of the budding yeast *Saccharomyces cerevisiae*, we identified a new mechanism for cellular diversification. We focused on studying the effect of drugs on ribosomal proteins, which are particularly sensitive to the effects of growth inhibition. We found that an ionic and osmotic stressor, lithium chloride, is able to elicit cell‐to‐cell expression heterogeneity in Rps22B, a ribosomal protein. The heterogeneity was present not only at the protein level, but also at the level of survival phenotype: while the low‐expression Rps22B subpopulation survived sustained stress better, cells with high Rps22B expression thrived in the absence of stress or after a brief period of transient stress. Additionally, we found an unexpected mechanism for establishing the observed heterogeneity ‒ the effect all but disappeared when an intron in the upstream untranslated region of the gene was deleted. Ribosomal proteins in yeast are enriched for the presence of introns, which were known to modulate gene expression levels of ribosomal proteins, but their role in cell‐to‐cell expression heterogeneity had not been observed. This drug‐induced effect was dose‐ and growth rate‐dependent, which possibly explains why it had been overlooked in previous approaches. Our results underscore the importance of studying the role of introns in establishing non‐genetic phenotypic heterogeneity.

The observed intron‐mediated heterogeneity in yeast has potential clinical implications. One advantage of using yeast is that, as a unicellular organism, it offers the possibility to quickly obtain a large clonal population containing many isogenic cells, which provides an excellent opportunity to study non‐genetic heterogeneity. However, another advantage of yeast is that despite being a unicellular organism, it exhibits gene homology with humans, hinting at research directions with potential clinical relevance. The human homolog of RPS22B, *RPS15a*, is a gene with multiple introns. A splicing mutation in *RPS15a* results in a congenital condition called Diamond‐Blackfan anaemia ‒ an erythroid lineage defect caused by an imbalanced production of ribosomes.^6^ Moreover, *RPS15a* has been implicated in colorectal, lung, glioblastoma, gastric and liver cancer.^7^ It is conceivable that the introns mediate phenotypic heterogeneity in tumours, and *RPS15a* would be an interesting starting point for testing this hypothesis.

More broadly, our study highlights the importance of studying drug‐induced heterogeneity in ribosomal proteins and other cellular components that are strongly affected by growth rate, in systems beyond the budding yeast. Just as yeast is able to harness induced heterogeneity to resolve the trade‐off between rapid growth and survival under stress, cancer cells face essentially the same trade‐off and might similarly diversify. This heterogeneity then affects the overall outcome of the treatment, which may work well in one subpopulation but fail in the other. Uncovering the mechanisms of non‐genetic diversity, and understanding how it interacts with genetic diversity,^8^ how it is induced by various drugs, and how we can prevent it or use it to our advantage is therefore instrumental for the advancement of therapies. While both genetic^9^ and transcriptional^10^ heterogeneity of cancer are intensively investigated and a connection between growth rate and chemosensitivity has been demonstrated,^11^ studies of drug‐induced heterogeneity in a setting that controls for growth rate of cancer cells are lacking. This limits our ability to characterise the drug effects on genes that are strongly dependent on growth rate, such as ribosomal protein genes, which may be critical for establishing phenotypically relevant heterogeneity. This is precisely why it will be worthwhile to adapt the isogrowth profiling approach, which relied on our ability to optically measure exponential growth rate in yeast cells in suspension, to the culture specifics required for mammalian cells, e.g. by using time‐lapse imaging to quantify the growth rate of cultured cells.

A promising aspect is that some of the limitations of isogrowth profiling in yeast may be more easily overcome in clinically relevant systems. Our original method uses single‐cell resolution to examine levels of individual proteins only, and not RNA, because single‐cell RNA sequencing in yeast involves the additional difficulty of lysing the cell wall. This hurdle is not present in mammalian cells. Thus, when adopted for isogrowth profiling, e.g. with cancer cells, existing droplet‐based single‐cell RNA methods^12^ could be used to study the influence of intronic elements on heterogeneity in a growth‐rate‐controlled manner. While conventional transcript‐end sequencing is not well suited for the study of RNA isoforms due to short reads not covering the entire transcript, recent progress has been made in this respect with full‐length transcript sequencing.^13^, ^14^, ^15^, ^16^ Since a large number of splicing factors and alternative splicing events have been shown to be involved in cancer processes^17^ and chemoresistance,^18^ time is ripe for uncovering the role of introns in inducing heterogenous drug‐responses, including in growth‐rate sensitive genes.

Isogrowth profiling is a method that uses combinatorial perturbations to eliminate an important confounding factor in the measurement of drug effects that is notoriously difficult to control during a perturbation ‒ the growth rate. Isogrowth profiling in its single‐cell flavour has proved its utility in discovering drug‐induced heterogeneity relevant to cell survival. Since many therapeutic interventions struggle when faced with heterogeneous cellular responses, isogrowth profiling should be adapted for clinically relevant model systems. When exploring the mechanisms underlying non‐uniform responses, the use of uniform inhibition may be just the way to go.
